# JAK Inhibitors for Myelofibrosis: Strengths and Limitations

**DOI:** 10.1007/s11899-024-00744-9

**Published:** 2024-10-14

**Authors:** K. Thaw, C. N. Harrison, P. Sriskandarajah

**Affiliations:** Department of Haematology, Guy’s Hospital, Great Maze Pond, London, SE1 9RT UK

**Keywords:** Myelofibrosis, Blood cancer, JAK-inhibitors, Toxicity

## Abstract

**Purpose of Review:**

The landscape of myelofibrosis (MF) has changed since the discovery of the *JAK2* V617F mutation and subsequent development of JAK inhibitors (JAKis). However, treatment with JAKis remain a challenge. In this review we critically analyze the strengths and limitations of currently available JAK inhibitors.

**Recent Findings:**

In MF patients, JAK inhibitors have been associated with reduced symptom burden and spleen size, as well as improved survival. However, durability of response and development of treatment resistance remain an issue. Recently, there has been increased efforts to optimize treatment with the development of highly selective JAK inhibitors, as well as use of combination agents to counter disease resistance through targeting aberrant signaling pathways.

**Summary:**

Treatment of MF patients with JAKi therapy can be challenging but the development of more potent and selective JAK inhibitors, as well as combination therapies, represent exciting treatment advances in this field.

## Introduction

The Philadelphia chromosome negative myeloproliferative neoplasms (MPN) are a group of onco-inflammatory conditions which involve aberrant activation of JAK-STAT pathway resulting in excessive and clonal proliferation of mature myeloid blood cells, and release of abnormal cytokines [1].

Myelofibrosis (MF) is a MPN characterized by bone marrow fibrosis, dyshemopoiesis, constitutional symptoms and extramedullary hemopoiesis. It can be classified as either Primary Myelofibrosis (PMF) or secondary through progression from Polycythemia vera (Post Polycythemia myelofibrosis; PPVMF) or Essential thrombocythemia (Post essential thrombocythemia myelofibrosis; PETMF) [2].

There has been a major change in the landscape of MPNs since the discovery of the activating V617F mutation in Janus kinase 2 (*JAK2*) in 2005 and the subsequent development of JAK inhibitors (JAKis) [3]. However, despite these advances, the management of MF with JAKis remains challenging due to limitations in efficacy, increased toxicity and development of treatment resistance.

### Pathophysiology of Myelofibrosis

In MF the most common driver mutations are in *JAK2*, *MPL* and *CALR* genes [4]. Generally, 65% of PMF patients carry the *JAK2* V617F mutation, while 20–30% have a *CALR* mutation and 5% have a *MPL* mutation [3–6]. By contrast in those with secondary MF, *JAK2* V617F is present in almost all cases of PPVMF and in 50% of PETMF, while *CALR* and *MPL* account for 30% and 10% of cases respectively [7].

**Fig. 1:**
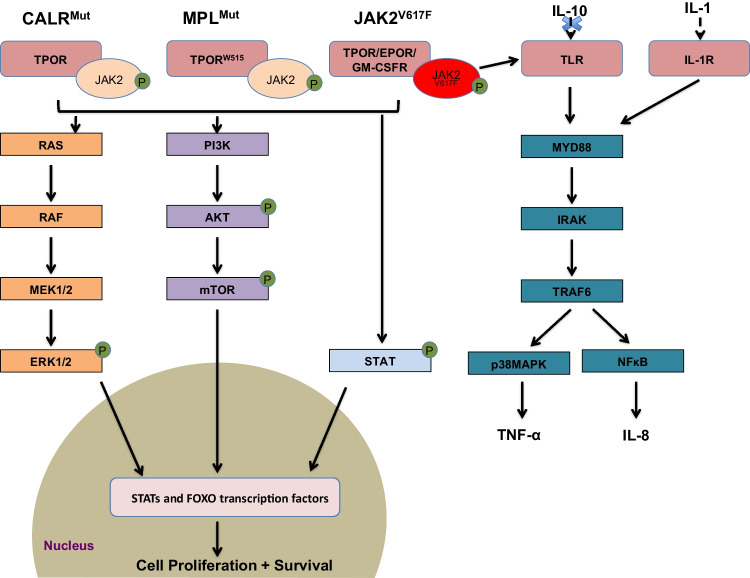
JAK2-related signaling pathways as well as associated inflammatory signaling commonly associated with MF. Mutations in CALR (CALRmut), MPL (TPORW515) and JAK2 (JAK2V617F) lead to the constitutive activation of JAK2-STAT as well as PI3K/AKT and MAPK/ERK which promote the transport of transcription factors (FOXO/STATs) to the nucleus. JAK2 also activated the Toll-Like receptor (TLR) pathway which affects the production of TNF-α and reduces its sensitivity to the anti-inflammatory cytokine IL-10. MPNs also secrete high levels of pro-inflammatory cytokines, including IL-1, which activates NFκB pathway. This in turn produces high levels of IL-8 which stimulates STAT3 and PI3K/AKT thus driving cell proliferation. Overall this highlights the cross-talk between various signaling pathways in myeloproliferative neoplasms

The pathophysiology for the development of this condition is through the JAK2-STAT pathway. Usually, JAK2 is activated by a variety of receptors including erythropoietin (EPOR), thrombopoietin (TPOR) and granulocyte/macrophage colony-stimulating factor (GM-CSF). Following stimulation, the JAK2-STAT pathway is activated, which then regulates the transport of transcription factors (STATs and FOXO) to the cell nucleus. In MF, JAK2-STAT signaling is constitutively activated by the driver mutations, causing excess cell proliferation, differentiation, and survival (Fig. 1) [8].

However, JAK2 also activates other major signaling pathways, including MAPK/ERK and PI3K/AKT. In the former, ERK is a key regulator of the cell cycle as well as multiple transcription factors while in the latter AKT inhibits apoptosis and activates translation via mTOR (Fig. 1) [8, 9]. Therefore, these pathways also act as drivers in MF.

As well as the above, JAK2 activates non-canonical signaling pathways including toll-like receptor (TLR) pathway which affects production of the inflammatory cytokine TNF-α (Fig. 1). In MF, *JAK2* V617F causes a defective negative regulation of TLR signaling resulting in the increased production of TNF-α and reduced sensitivity to the anti-inflammatory cytokine IL-10 [10].

Chronic inflammation is a characteristic feature of MPNs, including MF, and levels of inflammatory cytokines are typically increased in MPN patients [11]. Notably, JAK-STAT signaling is not the only contributor, as JAK2 inhibition has not been sufficient to normalize cytokine levels. The NFκB signaling pathway has since also been implicated in driving inflammation in these patients (Fig. 1) [12].

Overall, the pathophysiology of MF is far more complex than previously anticipated, with multiple signaling pathways implicated. Therefore, although JAK inhibition may be clinically effective, there is an increased risk of adverse events due to potential off-target effects. Importantly, by targeting specifically JAK2-STAT signaling, parallel pathways can potentially become overactivated thus driving drug resistance.

### Diagnosis and Risk Stratification of Myelofibrosis

The diagnosis and classification of MF has recently been updated and published by both the World Health Organization (WHO) and the International Consensus Classification (ICC), with the latter summarized in Table 1. In general, the 2022 WHO classification remains mostly unchanged compared with the fourth edition [2, 13] with only a minor change from palpable to splenomegaly detected on imaging. As noted in Table 1, those patients with PMF, the Dynamic International Prognostic Scoring System (DIPSS) as well as the DIPSS Plus (DIPSS +) incorporate clinical and cytogenetic features to risk stratify patients at any timepoint during the disease course [14, 15]. In secondary MF, a separate score was developed by Passamonti et al. [16] which was validated in 685 patients diagnosed with PPVMF and PETMF known as Myelofibrosis Secondary to PV and ET-Prognostic Model (MYSEC-PM) and is used in current clinical practice (Table 1) [16].

**Table 1:** ICC 2022 diagnostic criteria for both primary and secondary myelofibrosis as well as the prognostic models for risk stratification [13–16, 18–20]

Diagnostic criteria based on ICC 2022		
PMF	PPVMF	PETMF
Major Criteria (i) Megakaryocytic proliferation and atypia accompanied by reticulin and/or collagen fibrosis grades 2 or 3 (ii) *JAK2*, *CALR* or *MPL* mutation or presence of another clonal marker or absence of reactive myelofibrosis (iii) Diagnostic criteria for essential thrombocythemia, polycythemia vera, *BCR:ABL1*-positive chronic myeloid leukemia, myelodysplastic syndrome or other myeloid neoplasms are not met Minor Criteria • Anemia not attributed to a comorbid condition • Leukocytosis > 11 × 10^9^/L • Palpable splenomegaly • LDH level above reference range • Leucoerythroblastic Diagnosis requires all 3 major criteria and at least one minor criterion	Required Criteria (i) Previous established diagnosis of PV (ii) Bone marrow fibrosis of grade 2 or 3 Additional Criteria: (i) Anemia (*i.e.* below the reference range given age, sex, and altitude considerations) and a > 2 g/dL decrease from baseline hemoglobin concentration (ii) Leucoerythroblastic (iii) Increase in palpable splenomegaly of > 5 cm from baseline or the development of a newly palpable splenomegaly (iv) Elevated LDH level above the reference range (v) Development of any 2 (or all 3) of the following constitutional symptoms: > 10% weight loss in 6 mo., night sweats, unexplained fever (> 37.5 °C) Diagnosis of PPVMF is established by both required criteria and at least 2 additional criteria	Required Criteria (vi) Previous established diagnosis of ET (vii) Bone marrow fibrosis of grade 2 or 3 Additional Criteria: (iii) Anemia (*i.e.* below the reference range given age, sex, and altitude considerations) and a > 2 g/dL decrease from baseline hemoglobin concentration (iv) Leucoerythroblastic (viii) Increase in palpable splenomegaly of > 5 cm from baseline or the development of a newly palpable splenomegaly (ix) Elevated LDH level above the reference range (x) Development of any 2 (or all 3) of the following constitutional symptoms: > 10% weight loss in 6 mo., night sweats, unexplained fever (> 37.5 °C) Diagnosis of PETMF is established by both required criteria and at least 2 additional criteria
Prognostic models based on clinical features		
PMF	PPVMF/PETMF	
DIPSS	MYSEC-PM	
• Age > 65 years	• Age (0.15 × y of age)	
• Constitutional symptoms	• Constitutional symptoms	
• Hb < 10 g/dL	• Hb < 11 g/dL	
• WBC > 25 × 10^9^/L	• Blasts ≥ 3%	
• Blasts ≥ 1%	• Platelets < 150 × 10^9^/L	
	• Absence of CALR mutation	
Low (0): NR	Low (11): NR	
Int-1 (1–2): 14.2 years	Int-1 (11–13): 9.3 years	
Int-2 (3–4): 4 years	Int-2 (14–15): 4.4 years	
High (5–6): 1.5 years	High (> 16): 2 years	
DIPSS +		
• Age > 65 years		
• Constitutional symptoms		
• RBC transfusion need		
• Hb < 10 g/dL		
• WCC > 25 × 10^9^/L		
• Blasts ≥ 1%		
• Plt < 100 × 10^9^/L		
• Unfavorable karyotype		
Low (0): 15.4 years		
Int-1 (1): 6.5 years		
Int-2 (2–3): 2.9 years		
High (≥ 4): 1.3 years		
Prognostic models based on molecular/genetic markers		
MIPSS-70	MIPSS-70 +	GIPSS
• Constitutional symptoms	• Constitutional symptoms	• Very high risk karyotype**
• Hb < 10 g/dL	• Severe anemia (Hb < 8 g/dL in women; Hb < 9 g/dL in men)	• Unfavorable karyotype*
• WBC > 25 × 10^9^/L	• Moderate anemia (Hb 8–9.9 g/dL in women; Hb 9–10.9 g/dL in men)	• Absence of *CALR* type 1/like
• Blasts ≥ 2%	• Blasts ≥ 2%	• ASXL1 mutation
• Plt < 100 × 10^9^/L	• Absence of *CALR* type 1/like	• SRSF2 mutation
• Absence of *CALR* type 1/like	• 1 HMR included *U2AF1* Q157	• U2AF1 Q157 mutation
• 1 HMR	• ≥ 2 HMR included *U2AF1* Q157	
• ≥ 2 HMR	• Unfavorable karyotype*	
• Bone marrow fibrosis grade ≥ 2	• Very high-risk karyotype**	
Low (0–1): NR	Very low (0): NR	Low (0): 26.4 years
Int (2–4): 6.3 years	Low (1–2); 16.4 years	Int-1 (1): 8 years
High (≥ 5): 3.1 years	Int (3–4): 7.7 years	Int-2 (2): 4.2 years
	High (5–8): 4.1 years	High (≥ 3): 2 years
	Very high (≥ 9): 1.8 years	

*DIPSS* Dynamic International Prognostic Scoring System; *GIPSS* Genetically inspired prognostic scoring system; *Hb* Hemoglobin; *HMR* high molecular risk (one among *ASXL1, EZH2, SRSF2* or *IDH1/2*); *Int* Intermediate; *LDH* Lactate Dehydrogenase; *MIPSS* Mutation-Enhanced International Prognostic Scoring System; *Mo.* Months; *MYSEC-PM* Secondary to Polycythemia Vera and Essential Thrombocythemia Prognostic Model; *NR* Not reached; *Plt* Platelets; *RBC* Red blood cell; *WBC* White blood cell

*Chromosomal abnormalities except very high risk or sole 13q-, + 9, 20q-, chromosome 1 translocation/duplication or sex chromosome alterations including –Y

**Single or multiple abnormalities of -7, i(17q), inv(3)/3q1`, 12p-/12p11.2, 11q-/11q23, + 21 or other autosomal trisomies except + 8/9

In 2013, Vannucchi et al*.* discovered that ASXL1, SRSF2 and EZH2 mutations inter-independently predicted shorted survival [17]. This was later supported by Tefferi et al*.* which demonstrated that more than 80% of PMF patients harbored mutations in other myeloid genes including *ASXL1*, *TET2*, *EZH2*, *SFSF2*, *DNMT3A*, *U2AF1* and *IDH1/2* [18]. These mutations impacted overall and leukemia-free survival, independent of both the DIPSS + score as well as *JAK2/CALR/MPL* mutation status. Subsequently, prognostic scores were developed which incorporated these mutations and are summarized in Table 1 [19–21].

The main objective of these models are to facilitate treatment decisions, especially in MF patients eligible for allogeneic hematopoietic stem cell transplant (AHSCT). However, despite AHSCT being the only curative option, the morbidity and mortality risk remains high [22, 23]. Therefore, JAKis still have a major role in the treatment of MF.

### Management of Myelofibrosis

Based on the current National Comprehensive Cancer Network (NCCN) guidelines, management of MF is dependent on risk stratification as well as symptom burden [24]. The latter is usually assessed using the Myeloproliferative Neoplasm Symptom Assessment Form Total Symptom Score (MPN-SAF TSS) which is calculated as the mean score for 10 items including fatigue, bone pain and pruritus [25].

Overall, those patients with lower-risk MF (*i.e.* DIPSS ≤ 2; DIPSS +  ≤ 1; MYSEC-PM < 14) are managed according to their symptoms. If asymptomatic, the general recommendation is to observe, while those who are symptomatic should be commenced on cytoreductive therapy. Recommended options include Hydroxycarbamide, Pegylated Interferon alfa-2a or JAKis [24].

In those with higher-risk MF (*i.e.* DIPSS > 2; DIPSS +  > 1; MYSEC-PM ≥ 14), treatment is dependent on the platelet count and whether they are transplant-eligible or not. If patients are not transplant-eligible, or transplant is not feasible, JAKi therapy is recommended. The type of JAKi offered is dependent on their platelet count where with platelets ≥ 50 × 10^9^/L, options include Ruxolitinib, Fedratinib and Momelotinib. By contrast, with platelets < 50 × 10^9^/L, Momelotinib or Pacritinib are recommended [24].

### JAK Inhibitor Therapies

#### Ruxolitinib

Ruxolitinib is a potent and selective oral JAK1/JAK2 inhibitor and is currently approved for higher-risk MF patients as well as lower-risk patients with high symptom burden [23]. These recommendations were based on two pivotal studies, COMFORT-I and COMFORT-II which examined Ruxolitinib in int-2 and high-risk MF patients (Table 2) [26, 27].

**Table 2:** Summary of JAKis in MF including clinical trials

JAKi and primary targets	Clinical trials	SVR35 at 24 weeks	Summarized toxicities & limitations of JAKi
Ruxolitinib JAK1, JAK2	COMFORT-I (Ruxolitinib vs Placebo) COMFORT-II (Ruxolitinib vs BAT)	41.9% (Ruxolitinib) vs 0.7% (Placebo) 32% (Ruxolitinib) vs 0% (BAT)	• Grade 3/4 anemia • Grade 3/4 thrombocytopenia • Infection • Increased risk of NMSCs • Dose interruption/reduction
Fedratinib JAK2, FLT3	JAKARTA (Fedratinib 400 mg and 500 mg vs Placebo) JAKARTA-2 (Fedratinib 400 mg od escalating to 700 mg) second line FREEDOM second line (Fedratinib 400 mg monotherapy) FREEDOM 2 Fedratinib 400 mgs vs BAT	36% (Fedratinib 400 mg od) vs 1% (Placebo) 55% Fedratinib 25.7% Fedratinib 400 mg 35.8% Fedratinib 400 mg vs 6.0% BAT	• Grade 3/4 thrombocytopenia • Gastrointestinal toxicity • Wernicke’s encephalopathy
Pacritinib JAK2, FLT3, IRAK1	PERSIST-1 (Pacritinib vs BAT) PERSIST-2 (Pacritinib vs BAT) second line PAC203	19% (Pacritinib) vs 3% (BAT) 18% (Pacritinib 400 mg od) vs 3% (BAT)	• Diarrhea • Cardiac AEs
Momelotinib JAK1, JAK2, ACVR1	SIMPLIFY-1 (Momelotinib vs Ruxolitinib) SIMPLIFY-2 s line (Momelotinib vs BAT) MOMENTUM second line(Momelotinib vs Danazol)	26.5% (Momelotinib) vs 29% (Ruxolitinib) – non-inferior 7% (Momelotinib) vs 7% (Ruxolitinib) 23% (Momelotinib) vs 3% (Danazol)	• Gastrointestinal AEs • Peripheral neuropathy • Vitamin B1 deficiency

*AE* Adverse event; *BAT* Best available therapy; *JAKi* JAK inhibitor; *NMSC* Non-melanoma skin cancers; *OD* Once daily; *SVR35* Spleen volume reduction of 35%

Both COMFORT-I and COMFORT-II demonstrated that Ruxolitinib had superior efficacy in comparison to placebo in the former and best available therapy (BAT) in the latter [26, 27]. Regarding efficacy 41.9% of patients receiving Ruxolitinib demonstrated spleen volume reduction of more than 35% (SVR35) compared to 0.7% patients in the placebo group by week 24 in COMFORT-I [26]. Similar observations were made in COMFORT-II (28% Ruxolitinib *versus* 0% BAT) [27]. Of interest, a recent 5-year analysis of the COMFORT-II patients demonstrated nearly all of the Ruxolitinib cohort had some degree of initial SVR, which lasted for 48 weeks or more with continued therapy, and the median response duration was 3.2 years [28].

As well as spleen reduction, Ruxolitinib reduced symptoms with > 50% reduction in MF-SAF TSS observed in 45.9% of patients at week 24 compared to 5.3% on placebo in COMFORT-I [26]. This was also noted in COMFORT-II, using the FACT-Lym and EORTC QLQ-C30 scores [27].

As the *JAK2* V617F allele burden correlates with survival outcome in PMF, this was also examined in the recent 5-year analysis of the COMFORT-II study [27, 28]. At baseline 110 patients were *JAK2* V617F positive with median allele burden of 84%. Over the course of treatment with Ruxolitinib, one-third of evaluable patients had a > 20% reduction, which was sustained at week 192 [28].

As patients could cross over from the control arms in both COMFORT studies, survival benefit of Ruxolitinib was not easily demonstrated. Subsequently, Vannucchi et al. [29] published a pooled analysis across both COMFORT studies which accounted for this [29]. Overall, Ruxolitinib was associated with improved median overall survival (OS) compared to either placebo or BAT (5.3 years *versus* 3.8 years respectively). Furthermore, all patients who achieved SVR in the Ruxolitinib group had a better prognosis compared to those with either no change or an increase in spleen size [30].

However, several Ruxolitinib-associated adverse events (AEs) were reported in both COMFORT studies, the most common being anemia and thrombocytopenia [26, 27].

Anemia is a common finding in newly diagnosed MF patients and is a negative prognostic factor [30]. Therefore, Ruxolitinib can exacerbate this further, which can hinder the initiation and maintenance of this treatment in MF patients. In COMFORT-I and COMFORT-II, grade 3/4 anemia occurred in 45.2% and 42% patients respectively, although the rate of dose modification and/or interruption was low [26, 27].

Thrombocytopenia was another common AE observed with Ruxolitinib in both COMFORT studies [26, 27]. However, contrary to anemia, the degree of thrombocytopenia did limit Ruxolitinib dosing in MF patients [26, 27, 31]. To optimize dosing and titration of Ruxolitinib in MF patients with lower platelet counts, an open-label phase 2 study examined this further in patients with platelets between 50 to 100 × 10^9^/L. These patients were initiated on Ruxolitinib at 5 mg bid, which was titrated up to ≥ 10 mg bid by week 24 in 48% of patients. Notably, 73% of patients titrated to 10 mg bid achieved SVR35, while 44% achieved ≥ 50% reduction in MF-SAF TSS [32]. Similar findings were observed in the phase 3 JUMP study, with lowest dose 5 mg bid used in patients with platelets 50 to 100 × 10^9^/L [33]. EXPAND was another Phase 1b dose finding study that evaluated the starting dose of Ruxolitinib in 69 MF patients with baseline platelet counts of 50–99 × 10^9^ /L [34]. It had two strata: baseline platelet counts of 75–99 × 10^9^/L (Stratum 1) and baseline platelets of 50–74 × 10.^9^/L. Maximum safe starting dose was established in both strata as 10 mg twice daily and spleen response was achieved at week 48 in 33.3% and 30.0% of patients in Stratum 1 and 2 respectively [34].

However, the observational, real-world RUXOREL-MF study highlighted the importance of dose optimization to maximize Ruxolitinib clinical benefit [35]. This examined 209 MF patients treated with Ruxolitinib and focused on disease-related and treatment-related features in the first 6 months. Risk factors for shorter survival included ≤ 30% palpable spleen reduction as well as Ruxolitinib treatment at dose < 20 mg twice daily. Based on these findings, the group developed a prognostic model, the Response to Ruxolitinib after 6 months (RR6) score, which incorporated these factors as well as red cell transfusion requirement. Unsurprisingly, high-risk patients (*i.e.* score ≥ 2.5) had reduced median OS of 33 months compared to low-risk patients (score 0) where median OS was not reached [35].

Non-hematological AEs have also been observed with Ruxolitinib including infections and non-melanoma skin cancers (NMSCs). Regarding the former, a meta-analysis of clinical trial data from 6 randomized controlled studies did note an increased risk of herpes zoster in MF patients [36]. In relation to NMSCs, a 10-year retrospective cohort study of 564 patients, of which 188 were exposed to Ruxolitinib, demonstrated an increased incidence of NMSCs, especially squamous cell carcinomas [37]. Rampotas et al. [38] further supported this with a UK-wide retrospective study of MF patients who developed NMSC while on Ruxolitinib. They identified 106 NMSCs in 90 patients over a > 10 year period, with an increased risk of NMSC recurrence and metastasis noted in those who continued Ruxolitinib treatment [38].

Overall, Ruxolitinib is a highly effective treatment in MF patients as demonstrated by the COMFORT-I and COMFORT-II studies. However, the development of cytopenias, especially thrombocytopenia, can limit dosing and thus influence clinical outcome in these patients. Furthermore, discontinuation rates can be high ranging from 40 to 70% during the first year of treatment, and patient outcome after Ruxolitinib discontinuation is generally poor, with median progression-free survival (PFS) of 6.0 months and OS 11.1 months [39, 40].

#### Fedratinib

Fedratinib is a selective JAK2 and FMS-like tyrosine Kinase 3 (FLT3) inhibitor which was approved by the FDA for int-2 and high-risk MF patients who were Ruxolitinib naïve or resistant in 2019 [41]. These recommendations were based on results from the JAKARTA and JAKARTA-2 (Table 2). The former was a phase 3 placebo-controlled trial which evaluated once daily Fedratinib at 400 mg or 500 mg in JAKi-naïve MF patients [42]. Similar to Ruxolitinib, symptom responses at week 24 were observed in 34% of Fedratinib 400 mg patients compared to 7% placebo. Furthermore, up to 70% patients treated with Fedratinib experienced SVR at 24 weeks [42].

In JAKARTA-2, this was a phase II study which assessed the clinical activity of Fedratinib 400 mg daily in patients with int-2 or high-risk primary or secondary MF, previously treated with Ruxolitinib [43]. Notably, patients enrolled were originally classified as Ruxolitinib resistant or intolerant per investigator discretion. Furthermore, they had to have had at least 14 days of exposure to Ruxolitinib.

Initial analysis of JAKARTA-2 demonstrated increased efficacy with Fedratinib with improved spleen size and symptoms [43]. This analysis was recently updated using more stringent definitions of Ruxolitinib relapse, refractory or intolerance (*i.e.* Stringent Criteria Cohort). These criteria included relapse defined by Ruxolitinib treatment for ≥ 3 months with spleen regrowth, while refractory was defined by < 10% SVR from baseline at the same timepoint [44]. Overall 66 patients were included who received at least 6 cycles of Fedratinib. Responses were still maintained in this subgroup with SVR 30% and symptom response rate of 27% [44].

However, hematological AEs were reported in both JAKARTA and JAKARTA-2 including grade 3/4 anemia and thrombocytopenia [43]. Non-hematological AEs were also common, especially gastrointestinal toxicities [42, 43]. This was unsurprising given Fedratinib inhibits FLT3, and did result in dose interruption and reduction in 15% patients in JAKARTA-2 [43]. Importantly, Wernicke’s Encephalopathy (WE) was another potential AE related to Fedratinib and did result in this JAKi being placed on hold in 2013. However, a retrospective analysis did suggest that patients affected by WE had underlying predisposing conditions (*e.g.* malnutrition; gastrointestinal disturbance) [45]. Additionally, the prevalence of WE were lower than anticipated (0.4–0.7%). Based on these findings, the clinical hold was lifted in 2017.

Overall, Fedratinib is clinically effective and does reduce spleen size and symptom burden in patients previously treated with Ruxolitinib. However, the delivery of this drug is limited by both hematological and non-hematological AEs and requires close management. As a result, the FREEDOM and FREEDOM-2 studies were both designed with an aim to address this using prospective strategies to mitigate the gastrointestinal AEs and reduce risk of WE in int-2 and high-risk MF patients previously treated with Ruxolitinib [46, 47].

Results from the FREEDOM2 study were recently presented – this was a phase 3 randomized controlled trial with 201 patients of which 134 received Fedratinib while 67 received BAT [48]. Importantly, eligibility included a stringent definition for Ruxolitinib failure. Overall impressive responses were observed in those in the Fedratinib arm compared to BA, with higher SVF35 rates (35.8% *versus* 6% respectively) and symptom response (34.1% *versus* 16.9% respectively) [48].

#### Pacritinib

Pacritinib is a selective JAK2, FLT3 and interleukin-1 receptor-associated kinase 1 (IRAK1) inhibitor which was FDA approved in 2022 for int-2 and high-risk MF patients. This was based on the PERSIST-1 and PERSIST-2 studies which enrolled MF patients who were either JAKi-naïve in the former or been treated with 1 or 2 JAKis in the latter (Table 2) [49, 50]. MF patients enrolled in these studies had baseline platelets ≤ 100 × 10^9^/L.

Overall Pacritinib was clinically effective with higher SVR35 and symptom response observed in both studies compared to the control arms. Importantly, this benefit was observed in patients treated with prior Ruxolitinib and those with platelets ≤ 50 × 10^9^/L [51].

However, Pacritinib was placed on clinical hold in 2016 due to concerns over bleeding and cardiovascular events observed in the PERSIST-1 trial, and PERSIST-2 was abruptly closed [51]. To address this, a pooled retrospective efficacy analysis of 189 patients was conducted in 2021 [52]. This included all patients with severe thrombocytopenia (platelets < 50 × 10^9^/L) treated in the PERSIST-1 and PERSIST-2 studies. Overall, patients achieved significantly higher SVR35 and symptom response at week 24 compared to BAT [52]. Unsurprisingly, hematological AEs were grade 3/4, but did not result in dose reduction or discontinuation. Importantly, there were no excess hemorrhagic events compared to BAT (grade 3/4 13.6% *versus* 10.5% respectively) and no excess cardiac events (grade 3/4 9.1% *versus* 14% respectively) [52].

As fewer AEs were observed with the lower dose of Pacritinib, PAC203, a phase 2 study, evaluated this further in advanced MF patients [53]. Overall, modest SVR35 and symptom response rates were observed with Pacritinib 200 mg bid (9.3%) compared to 100 mg bid (1.8%) or 100 mg od (0.0%), although there were no excess grade ≥ 3 hemorrhagic or cardiac events [53]. A pooled safety analysis of patients with baseline platelets < 50 × 10^9^/L treated with Pacritinib 200 mg bid from PAC203 and PERSIST-2 reinforced these findings with no excess AEs reported [54]. Following these findings, Pacritinib was FDA-approved for treatment of MF patients with platelets < 50 × 10^9^/L.

Although Pacritinib is a viable therapeutic option in MF patients with profound thrombocytopenia, due to the initial hold of this agent and abrupt closure of PERSIST-2, the clinical benefit and safety still needs further clarification. This is currently being addressed in the PACIFICA study and results are awaited.

#### Momelotinib

Momelotinib is an inhibitor of JAK1 and JAK2, as well as Activin-A Receptor Type 1 (ACVR1) (Table 2). The ACVR1 receptor plays a key role in erythropoiesis as, when activated, this enhances hepcidin expression thus reducing iron availability for erythropoiesis. By inhibiting this receptor, alongside JAK1 and JAK2, Momelotinib suppresses hepcidin, thus potentially improving anemia in MF patients [55]. As mentioned earlier, anemia is a characteristic feature of MF and can be associated with adverse outcome [30]. Furthermore, JAKis to date were associated with worsening cytopenias, especially Ruxolitinib and Fedratinib [26, 27, 42, 43].

SIMPLIFY-1 and SIMPLIFY-2 were two randomized phase 3 trials conducted in patients with int-2 or high-risk PMF and secondary MF [56, 57]. In both trials patients were able to cross over to the Momelotinib arm after the 24-week response assessment. In SIMPLIFY-1, Momelotinib was evaluated against Ruxolitinib in JAKi-naïve MF patients. At baseline, 63.4% of patients in Momelotinib arm and 70.0% of patients in Ruxolitinib arm were transfusion independent. Unsurprisingly, a higher proportion of patients in the Momelotinib arm remained transfusion independent by week 24 compared to those treated with Ruxolitinib (66.5% *versus* 49.3% respectively) [56].

SIMPLIFY-2 evaluated Momelotinib against BAT, where MF patients had been exposed to Ruxolitinib for 28 days or more and either required red cell transfusions or dose reduction of Ruxolitinib due to grade ≥ 3 anemia/thrombocytopenia [57]. As expected, a higher proportion of Momelotinib patients remained transfusion-independent compared to BAT/Ruxolitinib at week 24 (43% *versus* 21% respectively) [57].

A key challenge in managing MF patients is maintaining the dose intensity of JAK inhibitors – this can be difficult, especially in those patients who develop cytopenias. As a result, Gupta et al. [58] performed a retrospective analysis of the SIMPLIFY studies and found that over 90% Momelotinib patients maintained dose intensity at 200 mg od throughout treatment compared to only 50% Ruxolitinib patients [58].

Another observation of Momelotinib in the SIMPLIFY studies was its limited myelosuppressive potential, especially thrombocytopenia [56, 57]. Furthermore, its efficacy for spleen, symptom and anemia response was maintained regardless of the platelet count. In a retrospective analysis by Kiladjian et al. [59], increased spleen and symptom response were observed with Momelotinib in 9.5% SIMPLIFY-1 and 44% SIMPLIFY-2 patients with platelets < 100 × 10^9^/L [59].

MOMENTUM was a recent phase 3 trial which compared Momelotinib against Danazol in int-2 and high-risk MF patients. Similar to the SIMPLIFY studies, a greater proportion of patients in the Momelotinib group achieved SVR35 at week 24 compared to Danazol (23% *versus* 3% respectively) as well as symptom responses (24.6% *versus* 9.2%) [60].

In 2023, Momelotinib was approved by the FDA for use in anemia patients with int-2 or high-risk MF. However, as expected, Momelotinib has been associated with non-hematological AEs. These were recently reviewed by Verstovsek et al. [60] who performed a pooled analysis of the SIMPLIFY and MOMENTUM studies [61]. The most common AEs reported were gastrointestinal, including diarrhea (26.8%), and peripheral neuropathy (14.8%). However, reassuringly, most AEs occurred during the first 24 weeks without cumulative toxicity and discontinuation rates were low [61].

### Upcoming Therapies

As demonstrated in the previous section, the JAKis have had a significant impact on MF management. It has been well established these drugs are able to induce spleen responses and reduce symptoms, with the added advantage of transfusion independence observed with Momelotinib. However, data comparing the long-term outcomes of these JAKis has been limited. A recent retrospective study addressed this by analyzing all JAKi-naïve MF patients treated with each of these JAKi in clinical trials between 2007 and 2013 [62]. Overall, 10-year survival rate was 16% which was similar across the different drug cohorts. Importantly, treatment discontinuation rates were high with less than 5% of patients remaining on long-term therapy, with the majority due to suboptimal response and disease progression [62]. These results highlighted that, despite recent advances, further work is needed to improve long-term outcomes in MF patients. We describe below upcoming therapies as well as novel combinations.

### Jaktinib

Jaktinib is a Momelotinib derivative, and is an inhibitor of JAK1, JAK2 and ACVR recently evaluated in int-2 and high-risk MF patients. Initial results from phase 2 trial were promising, with SVR35 observed in 54.8% patients on 100 mg bid at week 24 and ≥ 50% improvement in symptom score in 69.6% patients [63]. Recently, a phase 3 study comparing Jaktinib to Hydroxyurea (HU) demonstrated similar findings with SVR35 rates 72.3% in the former compared to 17.4% in the latter [64]. Importantly, less cytopenias were observed with Jaktinib and discontinuation rates were low.

### INCB160058

INCB160058 is a high affinity Pseudokinase (JH2) binding inhibitor of JAK2V617F that preserve cytokine dependent signaling while targeting cytokine independent activity of JAK2V617F. Pre-clinical studies in mouse models are promising with specific elimination of mutant JAK2*V617F* harboring cells with minimal impact on JAK2 wild type [65].

### CALR Therapies

As mentioned earlier, *CALR* is the second most common driver mutation detected in MF patients [7]. There are two variants of *CALR* mutation, of which type 1 is associated with longer survival compared to type 2, as well as less splenomegaly and lower platelet counts [66, 67]. Regardless of subtype, it has been observed that *CALR* mutant MF patients do not respond as effectively to JAKi compared to Ruxolitinib [68]. Therefore, new therapies have been developed targeting mutant CALR, including a monoclonal antibody and a mutant calreticulin peptide vaccine. These are both currently in trials though how success will be judged with these therapies is unclear and endpoints may need to be refined [69, 70]. Additionally, the bispecific-T-cell redirection antibody JNJ-88549968 is also under investigation with potential to achieve cures by eliminating MPN Clones [71].

### Combination Therapies

JAK2 is known to activate several pathways, (Fig. 1), including the PI3K/AKT/mTOR and RAS/RAF/MEK pathways. The latter pathway is of particular interest as *RAS* mutations with increased RAS/RAK/MEK signaling have been implicated in a diverse range of malignancies [72]. In MF specifically, *RAS* variants have been associated with advanced MF features, reduced 3-year OS and a higher incidence of progression to leukemia [73]. Furthermore, *RAS*-pathway mutations commonly emerge during Ruxolitinib treatment and correlate with reduced spleen and symptom response [74, 75]. Stivala et al. [76] explored this further *in vitro* where JAK2 inhibition suppressed MEK/ERK activation in MPN cell lines. However this was not observed in *JAK2*V617F mouse models *in vivo* and could be overcome with combined JAK/MEK inhibition with increased efficacy and reversal of fibrosis [76].

The PI3K/AKT/mTOR pathways are also activated by JAK2 and are constitutively activated in MPNs. Interestingly, Everolimus, an mTOR inhibitor, demonstrated clinical activity in MF patients in a phase 1/2 study [77]. Furthermore, *in vitro* studies demonstrated synergistic activity between Everolimus and Ruxolitinib in *JAK2*V617F mutant cell lines [78].

As mentioned, chronic inflammation is a hallmark characteristic of MF with the NFκB signaling pathway implicated in driving this. There has been increasing evidence of crosstalk between NFκB and JAK2-STAT signaling, and that the NFκB pathway drives MF progression [79]. Therefore there has been interest in targeting this pathway, especially in combination with a JAKi.

Bromodomain and extra-terminal (BET) family proteins (BRD2, BRD3, and BRD4) are epigenetic modifiers, with BRD4/BET implicated in NFκB-mediated inflammation [80]. Pelabresib (CPI-0610) is a selective oral BET inhibitor (BETi) which was recently investigated in combination with Ruxolitinib in JAKi-naïve MF. This combination was associated with durable improvements in spleen and symptom response, and was well tolerated [81].

## Conclusions

The JAKis have had a significant impact on the management of MF patients with the ability to reduce splenomegaly, improve symptoms and impact quality of life. However, there are ongoing challenges, with tolerance being a significant issue which affects dose intensity and thus outcomes in this challenging patient cohort. Furthermore, for those patients with resistant or refractory disease, options are limited and AHSCT is not an option for all due to the significant morbidity and mortality risk.

Upcoming JAKis demonstrate promise with the potential for better toxicity profile. However a major problem in the field is how these new therapies will be determined to be successful given the current reliance of the field on symptom benefit when this has not been linked to overall survival for patients. Spleen volume reduction is likely to remain a yardstick but emergent endpoints of interest are based around molecular data, cytokines, marrow morphology as assessed using artificial intelligence and perhaps inflammation [82]

In conclusion, there is still a need for further study of novel strategies in order to optimize outcomes in MF patients.

## Key References

- Köger N, Bacigalupo A, Barbui T, Ditschkowski M, Gagelmann N, Griesshammer M, et al. Indication and management of allogeneic haematopoietic stem-cell transplantation in myelofibrosis: updated recommendations by the EBMT/ELN International Working Group. Lancet Haematol. 2024;11(1): e62-e74.
  - ○ Recent EBMT guidelines for management of MF patients and those who should be referred for allogeneic transplant.
- Maffioli M, Mora B, Ball S, Iurlo A, Elli EM, Finazzi MC, et al. A prognostic model to predict survival after 6 months of ruxolitinib in patients with myelofibrosis. Blood Adv. 2022; 6(6):1855–1864.
  - ○ An important study which allows clinicians managing MF patients with Ruxolitinib to identify early those who may have poor overall outcomes and potential strategies to mitigate this.
- Rampotas A, Carter-Brzezinski L, Somervaille TCP, Forryan J, Panitsas F, Harrison C, et al. Outcomes and characteristics of nonmelanoma skin cancers in patients with myeloproliferative neoplasms on ruxolitinib. Blood. 2024; 143(2): 178–182.
  - ○ Although this is a retrospective study, this highlighted the increased risk of NMSCs and emphasized the importance of monitoring patients for this complication. Additionally, continuing Ruxolitinib in patients with known NMSC did increase the risk of relapse and metastasis, which will influence current practice.
- Venugopal, S, Mascarenhas, J. The odyssey of pacritinib in myelofibrosis. Blood Adv 2022; 6(16): 4905–4913
  - ○ This article gives an excellent overview of Pacritinib and the trial data which allowed this JAKi to gain approval in MF patients with thrombocytopenia.
- Gangat, N., Begna, K.H., Al-Kali, A. Hogan, W, Litzow, M, Pardanani, A, Tefferi, A. Determinants of survival and retrospective comparisons of 183 clinical trial patients with myelofibrosis treated with momelotinib, ruxolitinib, fedratinib or BMS- 911543 JAK2 inhibitor. Blood Cancer J. 2023; 13(3): 10.1038/s41408-022-00780-9
  - ○ This was the first retrospective analysis comparing all JAK inhibitors from recent trials and highlighted these agents had similar efficacy but responses were not sustained long-term.

## Data Availability

No datasets were generated or analysed during the current study.
